# A Critically Ill Influenza A-Positive Patient With Spontaneous Pneumomediastinum, Superimposed Bacterial Pneumonia, and Bilateral Pneumothoraces

**DOI:** 10.7759/cureus.57778

**Published:** 2024-04-07

**Authors:** Kirstin Acus, Stephen Meigher, Vinay Saggar

**Affiliations:** 1 Emergency Medicine, Columbia University College of Physicians and Surgeons, New York, USA

**Keywords:** acute cough, pneumothoraces, superimposed bacterial pneumonia, spontaneous pneumomediastinum (spm), influenza virus type a

## Abstract

Influenza most often causes a febrile viral syndrome inclusive of pulmonary irritation with cough, shortness of breath, and congestion. However, severe infection can also occur, causing significant viral pneumonia with Type 1 respiratory failure. and rare but life-altering complications such as pneumomediastinum, secondary bacterial pneumonia, acute respiratory distress syndrome (ARDS), viremia, and death. This was a case of a 20-year-old male with no significant past medical history who presented to the emergency department with shortness of breath and chest discomfort and was found to have Influenza A with Type I respiratory failure requiring High Flow Nasal Cannula (HFNC) and extensive pneumomediastinum, superimposed bacterial pneumonia, and bilateral pneumothoraces. It is possible that complications secondary to influenza A infections could be under-reported due to the extremely high prevalence of the viral infection in this country. In addition, complicated pneumomediastinum from Influenza infection is sparsely documented in young adult males and children, but its clinical course can be dramatic enough to include life-altering complications. This case should serve as a reminder to all emergency medicine providers that when evaluating unstable Influenza A patients, various tests should be considered on a case-by-case basis to risk-stratify the likelihood of emergent pathology.

## Introduction

Annual seasonal influenza epidemics have caused a predictable morbidity and healthcare strain across the world every winter season for the majority of modern history. During the 2023-2024 influenza season, it is estimated that 25 to 50 million infections occurred with up to 24 million healthcare visits, 670,000 hospitalizations, and somewhere between 17,000 to 98,000 deaths in the United States alone [1]. Influenza most often causes a febrile viral syndrome inclusive of pulmonary irritation with cough, shortness of breath, chest pain or congestion, as well as myalgias, malaise, and fatigue. However, severe infection can also occur causing significant viral pneumonia with hypoxemic respiratory failure and complications such as pneumomediastinum, secondary bacterial pneumonia, ARDS, viremia, and death [2]. This was especially noted in case reports during the H1N1 Influenza A swine flu epidemic and while rare, has been documented in pediatric cases [3-6].

Although rare and sparsely documented, these complications can often be dramatic enough to include life-altering complications, especially complicated pneumomediastinum [7-9]. This was a case of a 20-year-old male with no significant past medical history who presented to the emergency department with shortness of breath and chest discomfort and was found to have Influenza A with Type I respiratory failure requiring high-flow nasal cannula (HFNC) and extensive pneumomediastinum, superimposed bacterial pneumonia, and bilateral pneumothoraces.

## Case presentation

A 20-year-old male with no significant past medical history presented to the emergency department with the chief complaints of persistent dry cough, chest pressure, and shortness of breath. The patient reported that the symptoms first began as two days of a progressively worsening dry cough, as well as mild nasal congestion and isolated episodes of non-bloody, non-bilious emesis. The patient then awoke with severe chest discomfort and shortness of breath, after which he called emergency medical services (EMS). He reports not having received his influenza vaccine earlier in the year due to an unspecified allergy.

Vital signs were significant for the patient being febrile to 39.2 °C and tachycardic to 121 bpm. Pertinent physical exam findings included the patient seeming ill-appearing, diaphoretic, and selectively leaning forward in the stretcher throughout the evaluation. The patient was in mild respiratory distress with slight dyspnea while speaking and lung sounds were diminished in upper lung fields. 

**Figure 1:**
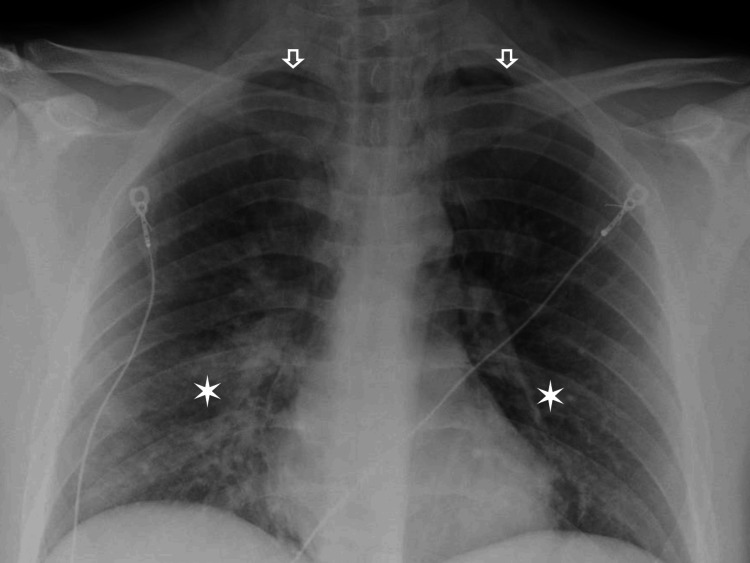
Upright Chest Xray The star notes the basilar opacities. The arrow indicates trace bilateral pneumothoraces

**Figure 2:**
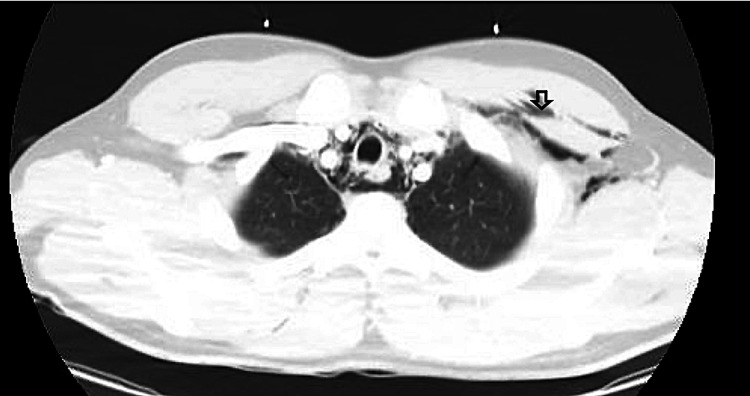
CT Chest with IV Contrast The arrow on the figure illustrates examples of cervical/upper thoracic scattered emphysema

**Figure 3:**
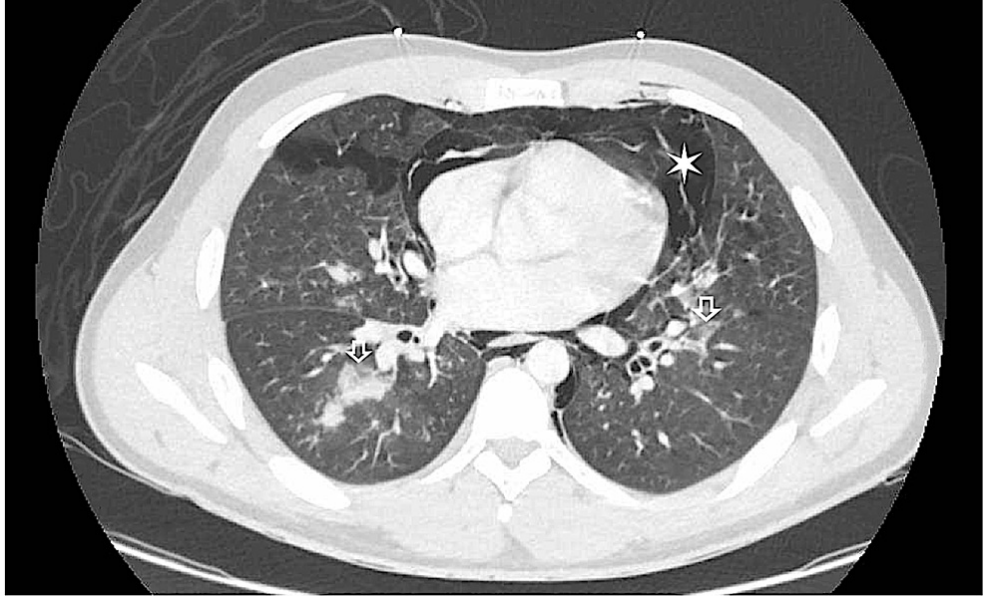
CT Chest with IV Contrast The star on the figure illustrates large-volume pneumomediastinum. The arrow shows the scattered patchy ground-glass consolidative opacities throughout the bilateral lungs concerning for pneumonia.

Labs were drawn and found to be significant for leukocytosis with a white blood cell count of 17 x 10^3^/uL, D-Dimer of 1,021 ng/ml DDU, a comprehensive metabolic panel within normal limits, and a respiratory panel positive for Influenza A. A portable chest x-ray showed bilateral opacities concerning for developing pneumonia (Figure 1), with a subsequent, emergent CT chest showed a large volume of pneumomediastinum, trace bilateral pneumothoraces, bilateral cervical and upper thoracic subcutaneous emphysema, and scattered opacifications (Figures 2, 3).

The patient was started on ceftriaxone and azithromycin for community-acquired pneumonia along with Tamiflu and admitted to the medical step-down unit for close monitoring with thoracic surgery consultation. The day after the initial presentation, the patient acutely decompensated requiring supplementary oxygen, which eventually escalated to 15L non-rebreather mask (NRB) followed by HFNC 50L/90% FiO2. The patient had an esophagogram done during admission which was negative for esophageal perforation and eventually was weaned to room air before being discharged 6 days after initial presentation back to home with pulmonary follow-up.

## Discussion

Influenza A represents the bulk of seasonal influenza infections during epidemics and thus the lion's share of morbidity, complications, and severity. Patients with severe influenza A infection develop lower respiratory illness syndromes with significant cough, dyspnea, and pain. Occasional case reports have documented severe and prolonged Influenza A infection causing bronchial obstruction or necrotizing pneumonia leading to small airway rupture and pneumomediastinum with ARDS in middle-aged and elderly adults [10]. Coughing may be severe and exertionally limiting, as well as lead to posttussive complications such as spontaneous pneumothorax, and rarely, spontaneous pneumomediastinum.

Spontaneous pneumomediastinum was first described in 1827 by Laennec and further described with subcutaneous emphysema in a postpartum patient by Hamman in 1939 with historical recognition of the condition and his eponymous moniker Hamman Sign, representing audible pericardial air ‘crunching’ with cardiac contractility on auscultation [11,12]. Pneumomediastinum occurs when intrathoracic pressure gathers significantly enough to rupture small airways and force pressurized air retrograde along the peribronchial and perivascular sheath to the mediastinum and the pericardial sac [13]. Pneumopericardium and pneumomediastinum are intra-thoracic diseases known to be more frequent in young and normally healthy patients, likely in part due to looser and more mobile peribronchial sheaths. Leading causes of rupture include severe coughing fits, bronchial obstruction, and necrotizing pneumonia, each leading to increased intrapulmonary pressure and small airway or alveolar rupture. Symptoms include persistent, severe cough, dyspnea, sharp chest pain, dysphonia, and neck swelling. Diagnosis may be captured on chest x-ray but the confirmatory study of choice is a non-contrast chest CT [13]. 

Supportive and conservative management is the mainstay of treatment in both severe influenza A and pneumomediastinum. Atmospheric air is absorbed from its small intrathoracic collections while ruptured small airways heal. Rarely, with high intrathoracic pressures or pericardial involvement, tension pneumomediastinum, pneumothorax, or tension pneumopericardium may develop. Treatment aims to resolve any life-threatening intrathoracic disease such as thoracostomy for a developing pneumothorax. Esophageal rupture should always be considered in cases of pneumomediastinum temporally related to vomiting. Very rarely, invasive procedures such as thoracostomy, sternotomy, and mediastinotomy are warranted [14]. The vast majority of the time, pneumomediastinum, subcutaneous emphysema, and pneumothoraces secondary to an influenza A infection are benign, self-limiting conditions. Appropriate treatment usually includes monitoring and supplementary O_2_ assistance as needed via nasal cannula or high-flow.

## Conclusions

Our patient was a rare case of an otherwise healthy young male with no pulmonary disease who endured significant complications from an acute Influenza A infection. It is possible that complications secondary to influenza A infections could be under-reported due to the extremely high prevalence of the viral infection in this country. The degree of pneumomediastinum found in this patient, as well as the delayed sequela and acute decompensation the patient experienced, should serve as a reminder to all emergency medicine providers that when evaluating these patients, various tests should be considered on a case-by-case basis to risk-stratify the likelihood of an emergent pathology.
